# Common grass blue butterflies (*Zizina otis*) are toxic throughout their geographic range

**DOI:** 10.1242/jeb.250745

**Published:** 2025-08-22

**Authors:** Marilia Fernandes Erickson

**Affiliations:** School of Natural Sciences, Faculty of Science and Engineering, Macquarie University, Sydney, NSW 2109, Australia

**Keywords:** Warning colour, Butterfly, Lycanidae, Climate zone, Sex differences, Aposematism

## Abstract

Aposematism consists of promptly identifiable signals paired with a secondary defence (e.g. toxins) and is an effective anti-predatory strategy. Visually aposematic signals are typically described as shades of red, yellow or white paired with black that generate conspicuous signals. Blue animals, however, are often overlooked and seldom tested for toxins even when conspicuous. Using *Daphnia* mortality assays, I show that the common grass blue butterfly, *Zizina oti*s, is toxic (∼40% fatality, while controls killed near 0%). Female *Z. otis* were more toxic than males, and tropical populations were more toxic than subtropical ones, with temperate populations being the least toxic. Population colour saturation and luminance, however, did not predict toxicity. My results demonstrate a literature bias in aposematic research and encourage the exploration of toxins in previously neglected taxa. Discovering more colour patterns associated with toxicity will help clarify how aversive learning works, and how signals that communicate unprofitability evolve.

## Teaser

**Summary:** The common grass blue butterfly is toxic in similar levels to known aposematic model species, exemplifying literature bias against blue toxic species.

## INTRODUCTION

Toxicity combined with conspicuous colour signals can effectively deter predator attacks through avoidance learning, a strategy known as aposematism (Roper and Wistow, 1986). Visually aposematic animals are often assumed to signal in shades of red and yellow, contrasted against black (Endler and Mappes, 2004). This assumption is supported by theoretical predictions that these colours are more stable under shifting environmental conditions (Arenas et al., 2014), and by innate aversion in bird predators (Lindström et al., 1999). While many known aposematic species such as *Heliconius* and *Ithominae* butterflies (Page et al., 2024) incorporate red, yellow and black into their wings, confirmation bias may occur if research only targets known toxic aposematic morphotypes. Indeed, studies that directly test whether blue species are aposematic are scarce (Umbers, 2013) despite examples of blue toxic species, such as the blue ring octopus (*Hapaloclaena* sp.) (Williams, 2010), poison dart frogs (*Dendrobates tinctorius*) (Barnett et al., 2018) and bees and wasps (e.g. *Eulaema*) (Cameron, 2004).

The perception of short wavelengths (blue) is widespread across the animal kingdom (Cashmore et al., 1999), including in predators (Umbers, 2013), unlike long wavelengths (red), which cannot be detected by most insect predators (van der Kooi et al., 2021). Birds are classic model predators in aposematic studies (Exnerová et al., 2007; Protti-Sánchez et al., 2023), yet insects can equally learn to avoid aposematic signals (Berenbaum and Miliczky, 1984; Raška et al., 2017) such that prey with blue aposematic signals could enjoy a selective advantage against insect predators.

Lycanidae butterflies, which are typically coloured blue, are predated by small arthropods (e.g. spiders, bees, dragonflies, beetles), birds and other small vertebrates (e.g. geckos, rats) (Alonso-Mejía and Marquez, 1994; Hendrick et al., 2022; Londt, 1999; Murata and Tsuchiya, 2017; Nacua et al., 2020). Yet, with few exceptions (New, 1993), they have not been considered to be toxic and aposematic. Common grass blues [Lycanidae, *Zizina otis* (Fabricius 1787)] are small grass-feeding butterflies, with adults often found in high abundance, fluttering close to the ground near food plants. Though aggregation is not unique to aposematic species, it is common among them; as such, *Z. otis* individuals have been reported to form colonies, swarm, engage in group migrations and even roost together in the evenings (Chang et al., 2020; Dunn, 2020). They feed on a wide variety of Fabaceae (Braby, 2016), which are a family rich in secondary metabolites (e.g. alkaloids and cyanogen) that are sequestered by other Lepidoptera (Nishida, 2002; Wink, 2013). In this study, I investigated whether *Z. otis* could be aposematic by testing its toxicity in standardised *Daphina* toxicity assays with individuals from different populations across a 2500 km gradient along the Australian East Coast. Specifically, I tested the following. (1) Whether *Z. otis* are more toxic in tropical or subtropical and temperate climates. I predicted that tropical butterflies are more toxic as a result of the higher abundance of metabolites in tropical plants (Coley and Barone, 1996; Rasmann and Agrawal, 2011). (2) Whether males and females differ in toxicity. I predicted that females are more toxic, because they are typically larger than males (Braby, 2016), and larger size has been related to the capacity to store toxins in frogs and ladybirds (Hagman and Forsman, 2003; Holloway et al., 1993; Phillips and Shine, 2006). (3) Whether populations that are more toxic differ in their coloration in order to signal greater toxicity. If *Z. otis* signals are honest, populations that have higher toxicity also would be more conspicuous (Arenas et al., 2015).

## MATERIALS AND METHODS

I collected *Zizina otis* from 11 different sites across the east coast of Australia: four sites in Sydney (temperate), four in Brisbane (sub-tropical) and three in Cairns (tropical). Butterflies were caught using an entomological net and kept in cooler bags until I reached the field base where they were euthanised by freezing (in a standard refrigerator, ∼0°C). They were kept frozen for as long as possible and transported in cooler bags to the laboratory, where they were kept in a −30°C freezer until experimentation. Individual butterflies were prepared for photography by clipping the wings off the butterfly body using sharp forceps. All photos were taken using a Sony A7 camera body (converted to full spectrum) with a Nikon EL-Nikkor 80 mm lens. I used the Badder IR-UV cut and Venus filters for RGB and UV photography, respectively. Photos included a 20% grey standard (Spectralon^®^) that I used to calibrate images in MICA toolbox (Troscianko and Stevens, 2015). I created multispectral images and modelled images of the forewing only to blue tit vision (luminance channel=double cone, receptor noise=0.05, Weber according to Hart et al., 2000). I used the luminance and saturation of the dorsal and ventral forewing as predictors, as *Z. otis* varied in their dorsal and ventral side but were uniform within sides. I measured 1–3 males and females of each population and averaged their colour metrics for males and females from each population. This was done because some of the individuals used in the toxicity assays had very damaged wings and I chose individuals in pristine condition for colour analysis. Additionally, some extracts contained more than one individual (see below), which would not allow for a single colour measure per toxicity trial.

For the toxicity analyses, I conducted 59 trials (20 subtropical, 21 temperate and 18 tropical), using 26 females and 33 males. Sample size was chosen to even out specimen number between sites. In sites where more butterflies were collected, I used R to select individuals randomly, aiming to keep a similar number of butterflies within sites (∼5) and sex (∼30 males and females). The wings were removed from butterfly bodies prior to extractions as bird predators typically detach the body from the wings prior to consumption of butterflies (Bowers et al., 1985; Orr, 2013). This is unlikely to affect butterfly toxicity, as several Lepidoptera species are known to store defensive fluids in their body (Brower and Glazier, 1975; Rojas et al., 2017; Whitaker et al., 2023). I extracted the toxins by mixing each butterfly body in 1 ml of methanol and ∼0.075 g of glass beads in a 1.5 ml tube. Tubes were placed in a tissue homogeniser (Precellys 24, Bertin Technologies) and subsequently centrifuged at 13,000 rpm for 10 min. After, I extracted the supernatant and evaporated the liquid using a vacuum concentrator (SP Genvac EZ-2.40 at 45°C for 2.5 h). Next, I re-hydrated each tube with MilliQ water to dilute each sample to a concentration of 0.008 g ml^−1^ of butterfly body; this was achieved by using the mass of the butterfly body to calculate the correct amount of MilliQ water to dilute the extract with and adding this to the solution using a precision pipette (Eppendorf). The concentration was decided upon weighing the first samples, but subsequently I collected smaller individuals from some of the populations and had to add two butterfly bodies (of the same sex and location) to reach the predetermined concentration. After extraction, solutions were kept in a refrigerator overnight and tested the next day. I added 10 *Daphnia magna* to 0.5 ml of butterfly stock and counted the number of dead *D. magna* after 4 h. To control for deaths due to the extraction procedure, and other, non-toxin biological molecules present in butterflies, I included extracts of a non-toxic butterfly, *Pieris rapae* (Müller et al., 2003) (*n*=10), MilliQ water (*n*=18) and methanol (*n*=18) (data from M.F.E., D. J. McLean and M. E. Herbertsein, unpublished).

**Fig. 1.**
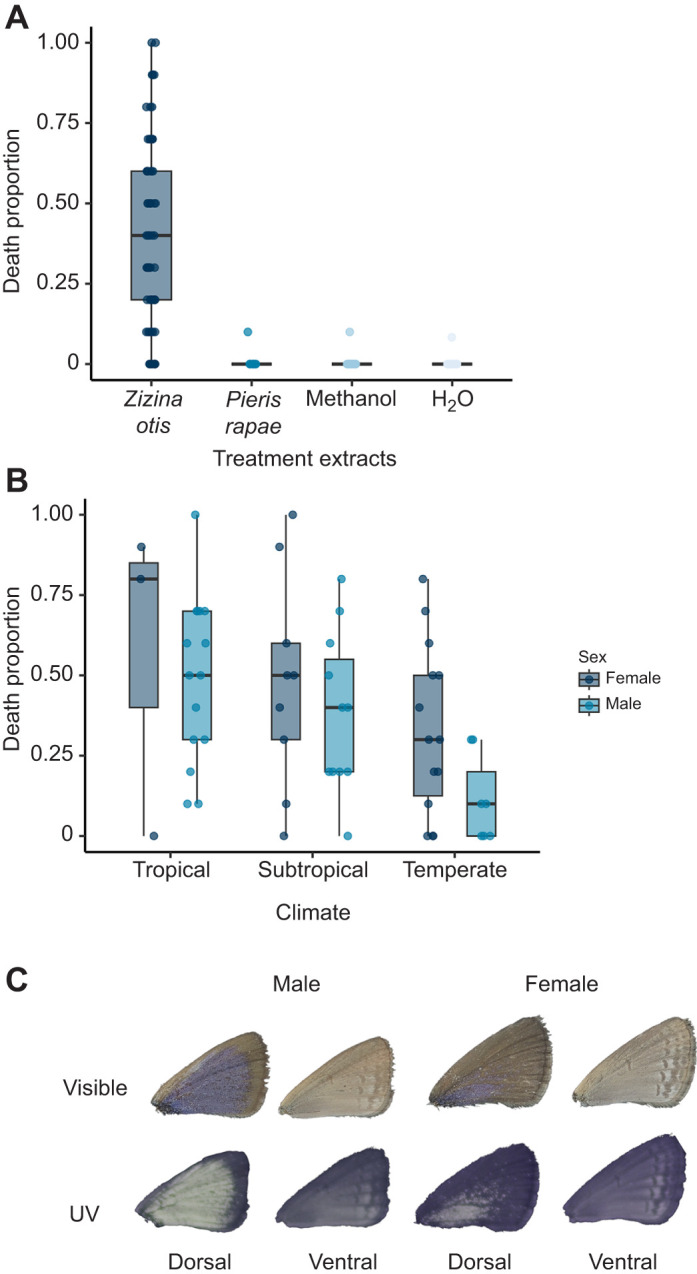
**Toxicity of common grass blue butterflies, *Zizina otis*.** Butterflies were collected in the field and taken to the laboratory for chemical extraction, where each trial consisted of 1 or 2 butterfly bodies added together. Toxicity was measured as the proportion of dead *Daphnia magna* after 4 h of exposure to *Z. otis* butterfly extract. (A) Boxplot comparison of the effect of *Z. otis* extract (*N*=59), *Pieris rapae* extract (a non-toxic butterfly; *N*=10), methanol (*N*=18) and water (*N*=18). The data for water, methanol and *P. rapae* extracts were not significantly different (logistic regression, *P*=0.98 and 0.67), but *Z. otis* was significantly different from water (logistic regression, *P*<0.0001). (B) Comparison of *Z. otis* from different climate zones (*N*=59 trials; tropical *n*=18, subtropical *n*=20 subtropical, temperate *n*=21; 26 females and 33 males). Tropical butterflies were most toxic and temperate butterflies were least toxic (logistic regression, *P*<0.05). For A and B, boxplots represent median proportion of death for the different treatments, boxes represent the 2nd and 3rd quartile and whiskers are the maximum and minimum value. (C) Example of male and female butterfly wings calibrated using 20% Spectralon^®^ standard; in UV photography, the lighter the region, more UV reflectance.

All analyses were done using R (http://www.R-project.org/). First, I compared mortality of extracts (*Z. otis* and controls) using a generalised linear model (glm). The effect of water was not significantly different from that of methanol (estimate±s.e.=0.022355±1.41812; *Z*=0.016; *P*=0.987) or *P. rapae* extracts (0.60338±1.41971; *Z*=0.425; *P*=0.671) but was different from that of *Z. otis* extracts (4.77979±1.00603; *Z*=4.751; *P*<0.0001) (Fig. 1A), so I proceeded with comparing intraspecific variation in *Z. otis* in mortality. I checked for collinearity of predictor variables using the ‘vif’ function from ‘car’ (Fox and Weisberg, 2019). Variables were not co-linear (<3) and were all included in the model (sex, climate, mean dorsal luminance, mean ventral luminance, mean ventral saturation and mean dorsal saturation). I used a binomial logistic regression where the response variable was *D. magna* death after 4 h of exposure to butterfly stock (count of how many died/survived after each trial, which is equivalent to the proportion of deaths). Site was originally included as a random variable but did not generate enough variation for a reliable model and was left out in the final model.

## RESULTS AND DISCUSSION

Luminance and saturation of dorsal and ventral wing sides were not significant predictors of toxicity (*P*>0.05) and were excluded from the final model, which included climate and sex. Tropical butterflies were more likely to be toxic (estimate±s.e.=0.51070±0.21761; *Z*=2.347; *P*=0.0189) than subtropical ones and temperate butterflies were the least likely to be toxic (−0.90873±0.22135; *Z*=−4.105; *P*=0.0000404). Males were less likely to be toxic than females (−0.62541±0.19821; *Z*=−3.155; *P*=0.0016) (Fig. 1B).

The work presented here is a large-scale test of toxicity of a small blue lycanid butterfly using a *Daphnia* toxicity assay. Whilst *Daphnia* are not predators, they are considered a good proxy for toxicity and have been used to quantify toxicity in multiple organisms (Arenas et al., 2015; Aslam and Nedvěd, 2024; Binns et al., 2025; Guilhermino et al., 2000; Harmon and Mousseau, 2009; Medina et al., 2020; Mora-Castro et al., 2021). Overall, the *Daphnia* assays suggest that *Z. otis* are toxic within the range of known toxic and aposematic insects such as ladybirds (Arenas et al., 2015). Because *Z. otis* are toxic and are easy to detect by humans (especially by their flight behaviour), they could be aposematic. Toxins can be stored for other functions, such as parasite deterrence (de Roode and Groot, 2024); therefore, testing for aversive learning with a relevant predator is needed to firmly conclude aposematism. Lycaenid butterflies contain many similar looking species, but so far only *Eumaeus* (a red taxon from the Theclinae sub-family) is known to sequester toxins (Rothschild et al., 1986). My results strongly encourage the exploration of toxicity in more lycaenids. An interesting first target is cycad-feeding species, for many butterflies (including *Eumaeus*) can sequester toxins such as cycasins or methylazoxymethanol from cycads (Bowers and Larin, 1989; Whitaker and Salzman, 2020). Furthermore, *Z. otis* is similar in colouration (grey ventral colour and blue dorsal colour) to many other members of the Polyommatinae subfamily, hinting at an undescribed mimicry ring.

Aposematism is often assumed to be size dependent with smaller species benefiting more from crypsis than from brightly coloured signals (Barnett et al., 2023). *Zizina otis* is one of the smallest butterfly species in Australia and, if confirmed to be aposematic, could be an interesting model to understand size limitation of aposematic prey, especially as smaller Australian butterflies appear to be more toxic (M.F.E., H. S. S. Daluwatta Galappaththige, D. J. McLean, J. Mappes, D. W. Kikuchi, H. M. Rowland and M. E. Herbersten, unpublished).

Tropical individuals were more toxic than sub-tropical ones and temperate butterflies were the least toxic. Toxin load is known to vary geographically (Ottocento et al., 2023), possibly as a result of the availability of host plants to sequester chemicals (Marsh et al., 1977; Speed et al., 2012). The tropics harbour a greater diversity and abundance of plant secondary metabolites (Coley and Barone, 1996), and butterflies can sequester different compounds that reflet local host plant chemical profiles (Jones et al., 2019). Additionally, predator dynamics can also influence the likelihood of chemical defences in aposematism species (Endler and Mappes, 2004). Tropical environments tend to have longer lived bird predators that emerge at a steady rate, which facilitates learning within a community (Kikuchi et al., 2021). This should drive the evolution and expression of aposematism in tropical prey communities where predator assemblages are more likely to learn and retain aversive interactions with aposematic prey (Kikuchi et al., 2021). Despite the toxicity results corroborating this hypothesis, we do not know whether common grass blue butterfly signals are learned by predators. Thus, investigating this pattern with a more diverse dataset is needed to understand how latitude can affect toxicity.

I did not find colour to be a good indicator of toxicity, despite the general assumption of aposematic signal honesty (Caro and Ruxton, 2019), because males were less toxic despite being more saturated in colour (Fig. 1C). These findings are, however, limited as I could not obtain colour and toxicity traits from the same individual (Caro and Ruxton, 2019), and toxicity and visual perception were tested using different model taxa (blue tit vision and *Daphnia* toxicity), which complicates the interpretation of signal honesty. My findings reported here should be corroborated with relevant predators, once they are known. Female *Z. otis* were more toxic than males, which is in accordance with other studies on toxic butterflies and beetles (Blount et al., 2023; Eggenberger and Rowell-Rahier, 1993; Marsh and Rothschild, 1974). As aposematic individuals gain protection while in motion (Speed and Ruxton, 2005), females might be slower flyers and are stationary during ovipositing, and would thus benefit from stronger toxins coupled with conspicuous aposematic signals (Ohsaki, 1995). I did not observe a dramatic difference in mobility between males and females, so it is unlikely that increased female toxicity is a response to their higher exposure. Both sexes were constantly flying and aggregating just above grass, which might amplify the signal in these small (wingspan ∼20 mm) butterflies. Alternatively, as toxin load can alter colouration (Blount et al., 2023), and male colouration is more heavily influenced by sexual selection (Kemp, 2007; Rutowski and Kemp, 2017; White et al., 2015), males may trade-off toxicity with stronger sexual signals.

My work suggests that common glass blues are a new exiting model species to study the function of toxins and evolution of aposematism. Although I have not yet demonstrated aposematism in common grass blues, these data suggest that common grass blue butterflies, because of their toxicity, could be somewhere on the evolutionary pathway to or from aposematism, which could be an equally exciting course of study. Not only are these butterflies broadly distributed and present in high densities but also they are highly variable in toxicity (from 0% to 100% *Daphnia* mortality) between populations, sexes and climate. The next steps in establishing whether *Z. otis* is indeed aposematic include chemical analysis of the toxin and its metabolic pathway, and testing predator aversion.
